# Marine Natural Product Inhibitors of Neutrophil-Associated Inflammation

**DOI:** 10.3390/md14080141

**Published:** 2016-07-26

**Authors:** Chun-Yu Chen, Yung-Fong Tsai, Wen-Yi Chang, Shun-Chin Yang, Tsong-Long Hwang

**Affiliations:** 1 Graduate Institute of Natural Products, School of Traditional Medicine, College of Medicine, Chang Gung University, Taoyuan 333, Taiwan; rainywoo2000@gmail.com (C.-Y.C.); yungfongt@gmail.com (Y.-F.T.); cweba7@gmail.com (W.-Y.C.); scyang6@vghtpe.gov.tw (S.-C.Y.); 2 Graduate Institute of Clinical Medical Sciences, College of Medicine, Chang Gung University, Taoyuan 333, Taiwan; 3 Department of Anesthesiology, Chang Gung Memorial Hospital, Taoyuan 333, Taiwan; 4 Department of Anesthesiology, Taipei Veterans General Hospital and National Yang-Ming University, Taipei 112, Taiwan; 5 Division of Natural Products, Graduate Institute of Biomedical Sciences, College of Medicine, Chang Gung University, Taoyuan 333, Taiwan; 6 Chinese Herbal Medicine Research Team, Healthy Aging Research Center, Chang Gung University, Taoyuan 333, Taiwan; 7 Research Center for Industry of Human Ecology and Graduate Institute of Health Industry Technology, College of Human Ecology, Chang Gung University of Science and Technology, Taoyuan 333, Taiwan

**Keywords:** marine natural products, neutrophils, anti-inflammatory, reactive oxygen species, formyl peptide receptor, phospholipase

## Abstract

Neutrophils are widely recognized to play an important role in acute inflammatory responses, and recent evidence has expanded their role to modulating chronic inflammatory and autoimmune diseases. Reactive oxygen species (ROS) and microbicidal compounds released from neutrophils that are recruited to the site of inflammation contribute to the pathogenesis of multiple inflammation-associated diseases such as chronic obstructive pulmonary disease, atherosclerosis, and hepatitis. Marine organisms are a valuable source of bioactive compounds with potential for industrial and pharmaceutical application. Marine natural products that inhibit neutrophil activation could be used as drugs for the treatment of inflammatory diseases. Numerous studies investigating marine natural products have reported novel anti-inflammatory agents. Nevertheless, the detailed mechanisms underlying their actions, which could facilitate our understanding of the molecular events occurring in neutrophils, have not been reported in most of the associated research studies. Therefore, in this review, we will present marine products that inhibit neutrophil-associated inflammation. Furthermore, we will be limiting the detailed discussion to agents with well-investigated molecular targets.

## 1. Introduction

Inflammation is a protective response initiated by activated immune cells and associated chemical mediators to counteract harmful stimuli such as pathogenic invasion, sterile injury, or irritants. Inflammation normally functions as a pivotal defense system for living organisms. Neutrophil granulocytes are the most numerous types of leukocytes (approximately 50%–70%) in mammals. They play a crucial role in the innate immune system and are the first effector cells to respond to chemotactic signals [1,2]. Activated neutrophils migrate to the site of infective or sterile inflammation where they perform various functions including phagocytosis, generation of reactive oxygen species (ROS), the release of proteolytic enzymes, and recruitment of other effector immune cells [2]. However, excessive neutrophil activation can cause prolonged self-induced damage and is involved in a variety of diseases such as cancer [3,4], respiratory disorders [5], liver diseases [6], inflammatory bone loss [7], and neurodegeneration [8]. Numerous studies have focused on exploring chemical or natural products that inhibit the activation of neutrophils to reduce excessive inflammation [9]. These research studies not only identified effective anti-inflammatory agents but also provided comprehensive analyses of the molecular targets of these compounds.

The origin and evolution of small organic molecules and creatures occur in the ocean, which covers approximately 70% of the earth’s surface. Since all organisms presently in existence have undergone intense evolutionary competition, it is reasonable to assume that marine organisms produce an enormous range of biologically active substances. Over the last decades, numerous marine natural products have been discovered with considerable pharmacological activities [10,11]. In particular, anti-inflammatory marine natural products have been the focus of intense interest due to their potential for use as therapeutic agents for inflammatory diseases.

This present report provides a systemic review of laboratory findings of investigated marine natural products and their derivatives with effects against neutrophil-associated inflammation. Although we are interested in all anti-inflammatory marine products, our particular focus will be on compounds with mechanisms of action that have been intensively investigated in neutrophils. We hope that this report will provide a useful foundation and source of data for further research into marine natural products with anti-neutrophil-induced inflammation potential. In addition, marine-based compounds may be tools for the elucidation of the mechanisms underlying neutrophil inflammatory responses.

## 2. Marine Natural Product Inhibitors of Formyl Peptide Receptor-1 (FPR-1) and Its Downstream Signaling Cascades

The formyl peptide receptor (FPR) family, which belongs to the G-protein-coupled receptor family, is one of the major mechanisms by which human neutrophils detect the presence of infective or sterile inflammatory signals. The FPR is a seven-transmembrane receptor with three loops exposed on the cell surface for ligand binding as well as intracellular loops for a series of signal transduction activities [12,13]. The human FPR family consists of the FPR1, FPR2/lipoxin A4 receptor (ALX), and FPR3, which all have pluripotent and diverse roles in the initiation, propagation, and resolution of inflammation [13,14]. FPR1 was the first identified and sequenced FPR in human neutrophils and is the predominant form in these cells. Various studies revealed that overwhelming activation of FPR1 on neutrophils induces severe inflammatory response syndrome and diseases [15,16]. The present review will refer to this FPR1 isoform only.

During infection, bacteria and affected host cells simultaneously release *N*-formylated peptides, the major FPR1 ligands [13,17]. The ligand interaction with FPR1 induces conformational changes in the receptor, causing G-proteins to dissociate into α and βγ subunits, which both activate a series of signal transduction pathways. There are numerous signaling pathways activated by Gα and Gβγ subunits, including the phosphatidylinositol-3-kinase (PI3K), Src-related tyrosine kinase, mitogen-activated protein kinase (MAPK), and phospholipase C (PLC). PLC cleaves the phospholipid phosphatidylinositol 4,5-bisphosphate (PIP2) into diacylglycerol and inositol 1,4,5-trisphosphate (IP3), which in turn releases Ca^2+^ from the endoplasmic reticulum and then activates protein kinase C (PKC). These molecular events induce a variety of functional responses including neutrophil chemotaxis, degranulation, and activation of nicotinamide adenine dinucleotide phosphate (NADPH) oxidase to produce ROS and transcriptional activity.

The possibility that FPR1, downstream intracellular signaling events, or both could be therapeutic targets has gained interest over the decades. Numerous efforts have made to discover marine natural compounds that antagonize FPR1 and its downstream pathway. Several marine FPR1 pathway antagonists with the potential for use as therapeutic agents for inflammatory diseases have been reviewed [18].

Following the identification of *N*-formyl-methionyl-leucyl-phenylalanine (fMLF) as a potent FPR1 agonist with high potency and efficacy in human neutrophils [19], fMLF was commonly used in experiments, which were aimed at the neutrophil inflammatory pathway. Table 1 lists the marine natural products that were reported to inhibit formyl-methionyl-leucyl phenylalanine (fMLF)-induced functional responses in neutrophils. However, most of their mechanisms of action have not been elucidated. In the following section, we will restrict our focus to the marine natural products that have had their molecular targets investigated.

### 2.1. IA-LBI07-1

Initially, the extract of the marine *Bacillus* sp., IA-LBI07-1 reported to inhibit superoxide generation and elastase release in fMLF-activated neutrophils [56]. Further elucidation of the underlying mechanism revealed that IA-LBI07-1 inhibited the binding of *N*-formyl-Nle-Leu-Phe-Nle-Tyr-Lys-fluorescein, a fluorescent analog of fMLF, to FPR1 in human neutrophils and FPR1-transfected HEK293 cells. These data provided strong evidence that IA-LBI07-1 is the only marine natural product that directly interferes with the binding of ligands to FPR1.

### 2.2. Staurosporine Aglycone

**Figure 1:**
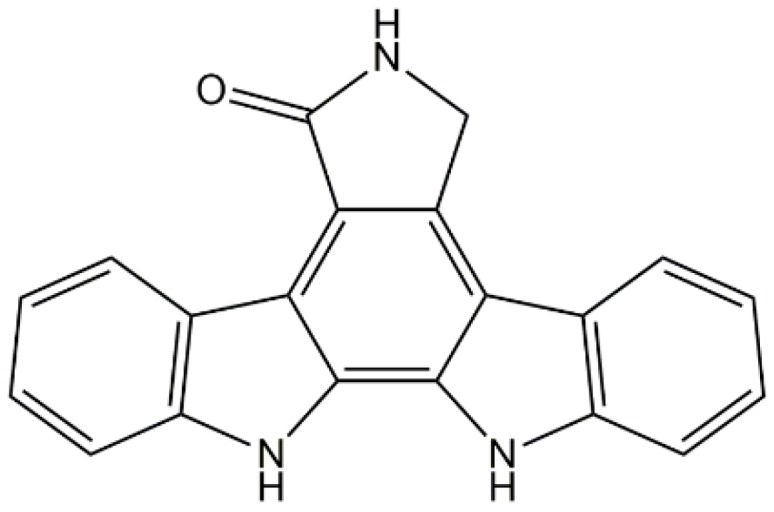
The structures of staurosporine aglycone.

Staurosporine, a natural product isolated from the bacterium *Streptomyces staurosporeus* [57], was discovered to have biological activities including antifungal and antihypertensive activities [58] and it non-selectively inhibited PKC. Substitution of one of the indole nitrogen atoms with non-glycosidic indolocarbazoles lacking an aminoalkyl side chain resulted in a staurosporine aglycone with increased selectivity for PKC. Horton et al. [59] isolated and identified the staurosporine aglycone (Figure 1) from a specimen of the marine ascidian, *Eudistoma* sp. They also found that the staurosporine aglycone inhibited the enzyme activity of seven of eight cloned PKC isoenzymes and inhibited oxygen release (detected by the presence of lucigenin) in neutrophils [59].

### 2.3. Secondary Metabolites of Marine Pseudomonas sp. (N11)

The secondary metabolites of a marine *Pseudomonas* sp. (N11) inhibited superoxide anion generation and elastase release in fMLF-activated human neutrophils, with half-maximal inhibitory concentration (IC_50_) values of 0.67 ± 0.38 and 0.84 ± 0.12 µg/mL, respectively [60]. Furthermore, a cell-free experimental system was used to verify that N11 had no ROS scavenging ability. It suppressed the phosphorylation of p38 MAPK, c-Jun *N*-terminal kinase (JNK), and calcium mobilization, but not extracellular signal-regulated kinase (ERK) and Akt in FMLP-induced human neutrophils. However, N11 failed to inhibit superoxide anion generation induced by phorbol myristate acetate (PMA), a specific PKC activator, suggesting that its inhibitory effect was not associated with the PKC pathway [60].

## 3. Marine Natural Product Inhibitors of Phospholipase A2 (PLA2) or Arachidonic Acid (AA) Metabolism

Phospholipase A2 (PLA2) is a lipolytic enzyme found in all types of mammalian cells as well as insect and snake venom [61,62]. PLA2 isoforms are subdivided into several protein families with common enzymatic activity. Secreted and cytosolic PLA2 (sPLA2 and cPLA2, respectively) are the two most notable PLA2 families expressed in neutrophils [62]. The sPLA2 family includes low-molecular-weight and Ca^2+^-dependent extracellular enzymes while the enzymes of the cPLA2 family are significantly larger. PLA2 hydrolyzes the glycerophospholipids at the sn-2 position [63], which releases AA and lysophospholipids. AA is oxygenated by 5-lipoxygenase (5-LO) and cyclooxygenase (COX) to produce 5′-hydroperoxyeicosatetraenoic acid (5-HPTETE) and prostaglandin H (PGH2), respectively. 5-HPTETE is the unstable precursor of 5-hydroxyicosatetraenoic acid (5-HETE), which is successively converted to leukotriene A4 (LTA4) and then leukotriene B4 (LTB4), a potent chemoattractant of leukocytes. PGH2 is converted to PGD2, PGE2, PGI2, and thromboxane A2 (TXA2) [62,64]. These PLA2-related chemical mediators are referred to as eicosanoids and play a critical role in virtually every step of the host defense and inflammatory response. Moreover, the molecular events following FPR1 activation are known to be involved in activating PLA2. Cytosolic PLA2 has been shown to be activated by the major molecular events downstream of FPR1 including activation of MAPK [65,66,67], intracellular Ca^2+^ [67,68], and PKC [69] signaling.

Since the discovery of manoalide, which is the first identified marine natural product with PLA2 inhibitory activity, various marine PLA2 inhibitors have been discovered and reviewed in detail in previous articles [70,71]. Here, we will further discuss the marine-based inhibitors of PLA2 or its downstream pathway in neutrophils that were not mentioned in previous reviews.

### 3.1. Avarol, Avarone, and Their Derivatives

**Figure 2:**
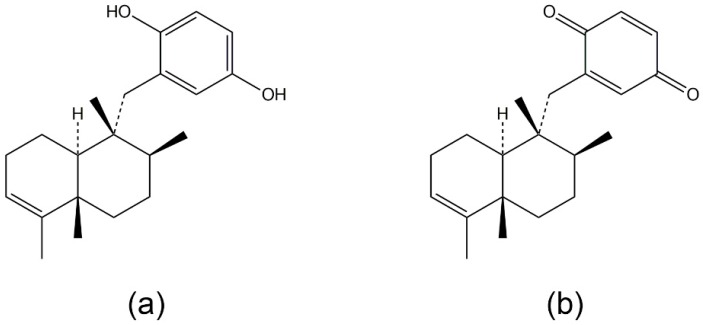

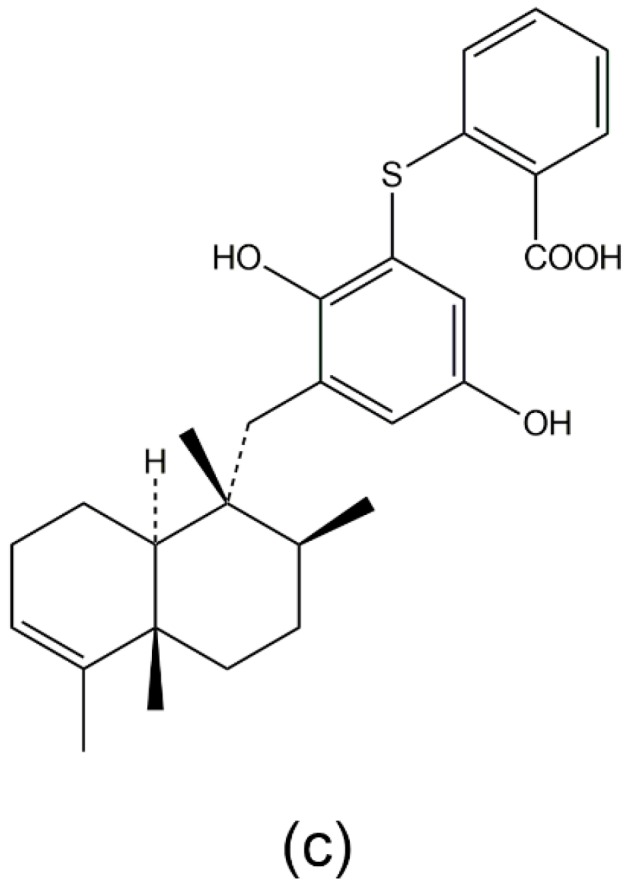
The structures of: avarol (**a**); avarone (**b**); and avarol-3′-thiosalicylate (**c**).

Avarol and avarone (Figure 2a,b, respectively) are sesquiterpenoid hydroquinones isolated from the marine sponge *Dysidea avara* [72]. Avarol is a cytostatic agent with potent antileukemic activity [73], and it also displays antibacterial and antifungal activities against a limited range of microorganisms. Both avarol and avarone suppressed superoxide generation stimulated by fMLF or PMA in rat peritoneal leukocytes, which consist of approximately 80% neutrophils. They also reduced LTB4 and TLXB2 release induced by a calcium ionophore (A23187) in rat peritoneal leukocytes. In vivo studies revealed that both substances potently inhibited carrageenan- and PMA-induced murine paw and ear edema, respectively [72]. However, only avarol inhibited the activity of human recombinant synovial PLA2 directly [72]. In addition, the avarol derivative, avarol-3′-thiosalicylate (TA; Figure 2c) inhibited PMA-stimulated ROS generation in human neutrophils [74,75]. TA also inhibited LTB4 generation in human neutrophils stimulated with a calcium ionophore (A23187), as well as the activity of the human synovial recombinant sPLA2. These data suggest that avarol, avarone, and the derivative TA, are inhibitors of the PLA pathway in neutrophils.

### 3.2. 2-Octaprenyl-1,4-hydroquinone, 2-[24-Hydroxy]-octaprenyl-1,4-hydroquinone, 2-Prenyl-1,4-hydroquinone, 2-Diprenyl-1,4-hydroquinone, 2-Triprenyl-1,4-hydroquinone, and 2-Tetraprenyl-1,4-hydroquinone

**Figure 3:**
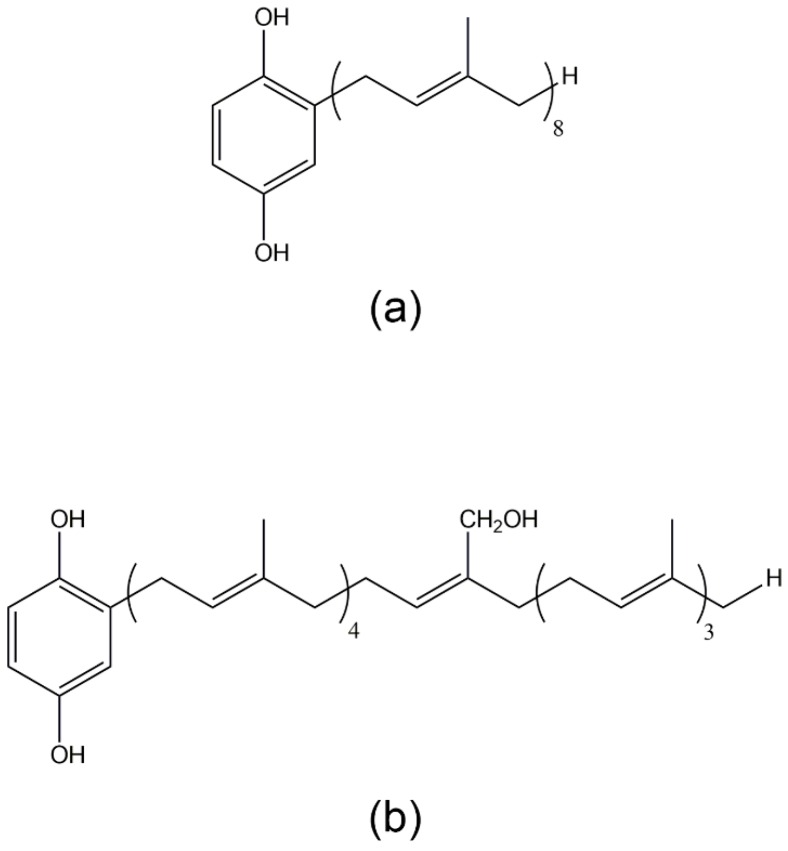

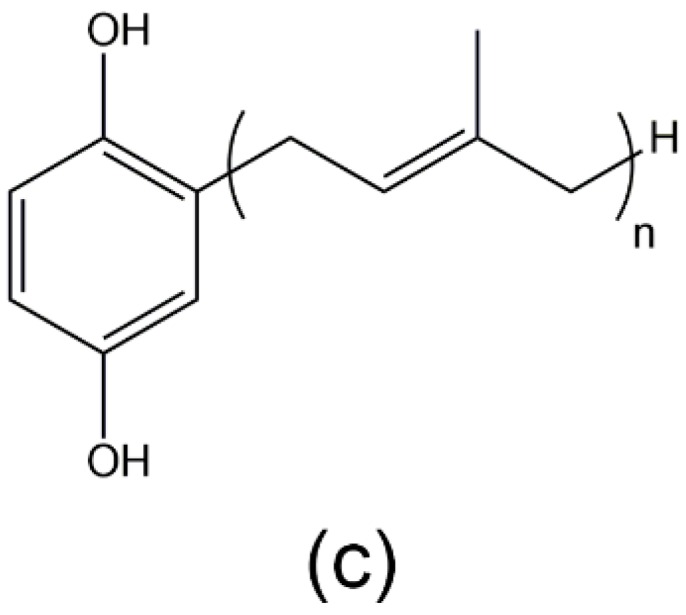
The structures of: IS2 (**a**); IS3 (**b**); and prenyl-hydroquinones (H1: *n* = 1; H2: *n* = 2; H3: *n* = 3, H4: *n* = 4) (**c**).

Studies of two 2-polyprenyl-1,4-hydroquinone derivatives, 2-octaprenyl-1,4-hydroquinone and 2-[24-hydroxy]-octaprenyl-1,4-hydroquinone (IS2 and IS3, respectively; Figure 3a,b), and four prenyl-hydroquinone derivatives, 2-prenyl-1,4-hydroquinone, 2-diprenyl-1,4-hydroquinone, 2-triprenyl-1,4-hydroquinone, and 2-tetraprenyl-1,4-hydroquinone (H1, H2, H3, and H4, respectively; Figure 3c), isolated from the sponge *Ircinia spinosula* revealed their inhibitory effects on PLA2 activity in human neutrophils [76,77]. Both IS2 and IS3 reduced ROS generation and degranulation in human neutrophils stimulated with PMA. They decreased the synthesis and release of TXB2 and LTB4 in a mixed suspension of human neutrophils and platelets stimulated by the ionophore A23187. In animal studies, IS2 and IS3 showed topical anti-inflammatory activity against PMA-induced ear inflammation in mice. In another study, H1, H2, H3, and H4 scavenged ROS and inhibited 5-LO activity in human neutrophils induced by PMA [76]. The inhibition of 5-LO activity was also demonstrated in an air pouch animal model. These studies revealed that differences in the lateral chain of the prenyl-hydroquinones critically modified their anti-inflammatory activity. They also provide an example of how the modification of known natural products may offer a promising strategy for modulating the anti-inflammatory effect of drugs.

### 3.3. Lyprinol

Lyprinol is a freeze-dried, stabilized, green-lipped mussel (*Perna canaliculus*) powder, which is used as a nutritional supplement to ameliorate signs of inflammation. An in vitro study revealed that four of the lyprinol subfractions containing polyunsaturated acids with four, five, and six double bonds inhibited the transformation of added arachidonate to LTB4 and 5-HETE in neutrophils stimulated by A23187 [78].

### 3.4. Ircinin

**Figure 4:**
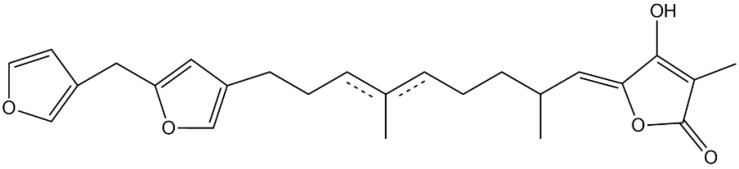
The structure of ircinin.

A mixture of the C25 furanosesterterpene tetronic acid geometrical isomers ircinin-1 and ircinin-2, tentatively named ircinin (Figure 4) isolated from sponges of the *Sarcotragus* sp. directly inhibited *Naja naja* venom, human synovial recombinant, bee venom, and zymosan-injected rat air pouch sPLA2 activities. Furthermore, ircinin dose-dependently inhibited PMA-stimulated superoxide generation and elastase release stimulated by fMLF. Moreover, ircinin suppressed the release of LTB4 in A23187-induced human neutrophils [79].

### 3.5. Dihydroxyicosanoids

Three dihydroxyicosanoids, 12(*R*),13(*R*)-dihydroxyicosa-5(*Z*),8(*Z*),10(*E*),14(*Z*)-tetraenoic acid, 12(*R*),13(*R*)-dihydroxyicosa-5(*Z*),8(*Z*),10(*E*),14(*Z*),17(*Z*)-pentanoic acid, and 10(R*), 11(R*)-dihydroxyoctadeca-6(*Z*),8(*E*),12(*Z*)-trienoic acid, were isolated from the marine alga *Farlowia mollis*. These marine products inhibited ROS generation in fMLF-activated human neutrophils and modulated 5-LO activity in human neutrophils stimulated with A23187 [39].

## 4. Marine Natural Products that Regulate Phosphorylation of Intracellular Mediators

**Figure 5:**
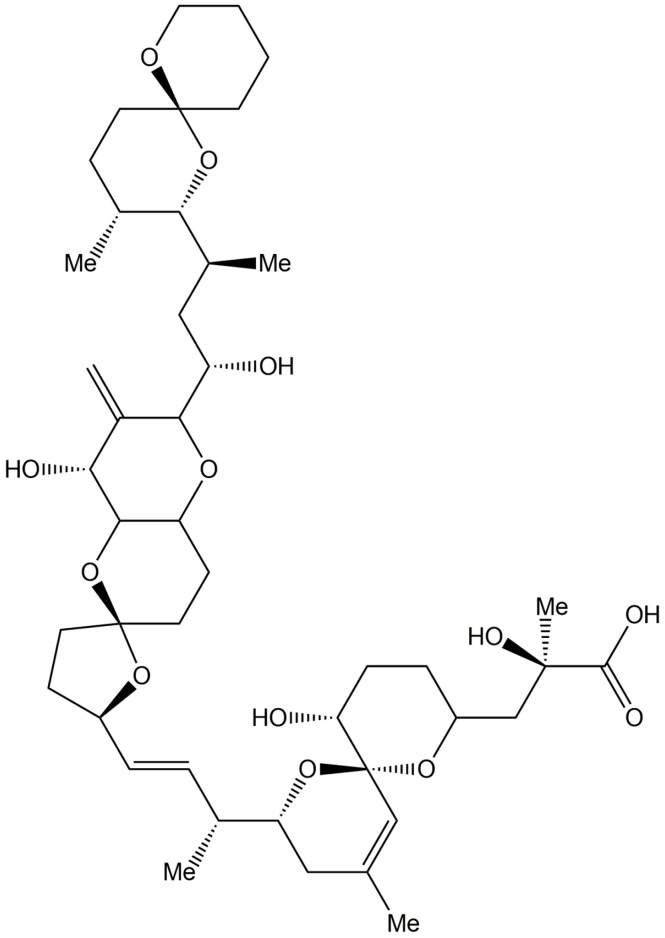
The structures of okadaic acid.

Okadaic acid (OKA, Figure 5), a toxin produced by several spp. of dinoflagellates, is a potent inhibitor of serine/threonine protein phosphatase (PP) type 1 (PP1) and type 2A (PP2A), which inhibit the dephosphorylation of proteins in numerous cells. OKA displayed contrasting effects on ROS generation in human neutrophils. OKA enhanced ROS generation in fMLF-stimulated neutrophils by inhibiting PP1 and PP2A [80,81,82]. Since OKA increases protein phosphorylation by inhibiting serine/threonine PPs, it is speculated that the enhanced fMLF-induced ROS generation by OKA was due to prolonged protein phosphorylation, which increased kinase activity and, thus, delayed the termination of the respiratory burst. On the other hand, pretreatment with OKA reduced PMA-stimulated superoxide generation [81,82,83]. In addition to its role as a serine/threonine phosphatase inhibitor, previous studies revealed that OKA increased tyrosine phosphatase activity and, thereby decreased tyrosine phosphorylation [83,84]. This might explain the mechanism by which OKA inhibited the PMA-induced respiratory burst. Moreover, OKA also increased the maximal rate and duration of superoxide release in response to arachidonate; however, OKA alone did not induce any release of superoxide [85]. Taken together, these studies using OKA, a thoroughly investigated marine natural product, revealed that phosphatases could positively or negatively affect the intensity of the respiratory responses induced by fMLF or PMA, respectively. These studies on OKA also provide examples of intensively investigated marine natural products that could be used as effective tools for the in-depth study of cellular inflammatory molecular mechanism.

## 5. Marine Natural Product Inhibitors of Neutrophils Migration and Adhesion

Following inflammation, circulating neutrophils are attracted by a precisely regulated sequential response induced by an increasing gradient of chemoattractants. The neutrophil extravasation is a complex process, and various molecules are involved. Briefly, as resident sentinel macrophages recognize invading pathogens, they release inflammatory mediators such as IL-1, TNF-α and C5a. These mediators upregulate the expression of P-selectin and E-selectin on the surface of endothelium. The binding of glycosylated ligands of neutrophil to the selectin of the endothelium tethers the rolling neutrophils. For tight adhesion, the rolling neutrophils are further activated, and the surface low-affinity integrin molecules were switched to a high-affinity state. To leave the vasculature, neutrophils have to transmigrate the endothelium. Transmigration requires multiple adhesion molecules, such as integrins, intercellular adhesion molecules, vascular cell adhesion protein 1, CD31, CD99, junctional adhesion molecules, and epithelial cell adhesion molecule [1]. Subsequently, the cytoskeletons of neutrophils are reorganized that neutrophils extend pseudopodia and pass through endothelial gaps. Finally, neutrophils have to move through the basement membrane, which was accomplished by digesting basement membrane with releasing proteases.

Stimuli targeting various intracellular signaling pathways such as Ca^2+^, MAPK, and PLA2 induced the activation and migration of neutrophils. Most marine natural products that were mentioned in the previous sections might also regulate neutrophil migration. In the following section, we will review studies involving experiments that described additional marine products that inhibited the migration of human neutrophils. Inflammatory cell migration is a complex process that involves a variety of immune and non-immune cells. The molecular mechanisms underlying the action of migration inhibitors not only target neutrophils but also target other inflammatory cells and chemical mediators.

### 5.1. Polysaccharides

Polysaccharides (PLS) extracted from substances of marine origin have shown several biological activities such as anti-inflammatory, antinociceptive, and anticoagulant [86,87,88,89]. A sulfated PLS fraction extracted from the alga *Hypnea musciformis* [86], reduced the inflammatory response and neutrophil migration, which appeared to be mediated by the nitric oxide (NO) signaling pathway. Regular and sulfated PLS extracted from *Digenea simplex* [88] and the algae *Gracilaria caudate* [87], respectively, reduced neutrophil migration by decreasing the production of pro-inflammatory cytokines. Moreover, a fucosylated chondroitin sulfate (fCS) extracted from the sea cucumber *Holothuria forskali* is composed of repeated trisaccharide units, and its poly- and oligosaccharides inhibited human neutrophil elastase activity and the migration of neutrophils through an endothelial cell layer by inhibiting selectin interactions [90].

### 5.2. Lectins

Lectins are ubiquitous proteins that bind specifically and reversibly to carbohydrate structures, particularly the sugar moiety of glycoconjugates and glycoproteins. One of the major functions of lectins in animals is to facilitate cell-cell contact. Marine algal lectins have lower molecular weights than those found in other organism and, therefore, have attractive biological applications. For example, small algal lectin molecules may be less antigenic than the larger plant lectins are [91]. Moreover, their structures contain several disulfide bonds that provide high thermostability [92]. Some lectins extracted from marine alga including *Hypnea cervicornis* agglutinin (HCA) isolated from the alga *H. cervicornis* [91], *Pterocladiella capillacea* lectin (PcL) from the red alga *P. capillacea* [93], *Caulerpa cupressoides* lectin (CcL) from the green alga *C. cupressoides* [94], and *Holothuria grisea* agglutinin (HGA) isolated from *Holothuria grisea* [95] were reported to have antinociceptive effects in murine inflammatory pain studies. The antinociceptive effect of these marine lectins was associated with reduced neutrophil recruitment to the inflammatory tissue. Furthermore, studies of HCA and HGA found that the mechanism underlying the antihypernociceptive activity of HCA and HGA is mediated by increased NO production. In contrast, some other marine-based lectins showed chemoattraction. Nattectin, a C-type lectin isolated from the venomous fish *Thalassophryne nattereri*, was investigated using a murine model of inflammation [96]. Neutrophils were recruited to the mouse footpad, which was injected with nattectin. Moreover, the levels of the chemoattractants including the lipid mediator LTB4, pro-inflammatory cytokine interleukin (IL)-1b, and chemokine KC were increased at the site of nattectin injection.

### 5.3. Miscellaneous

**Figure 6:**
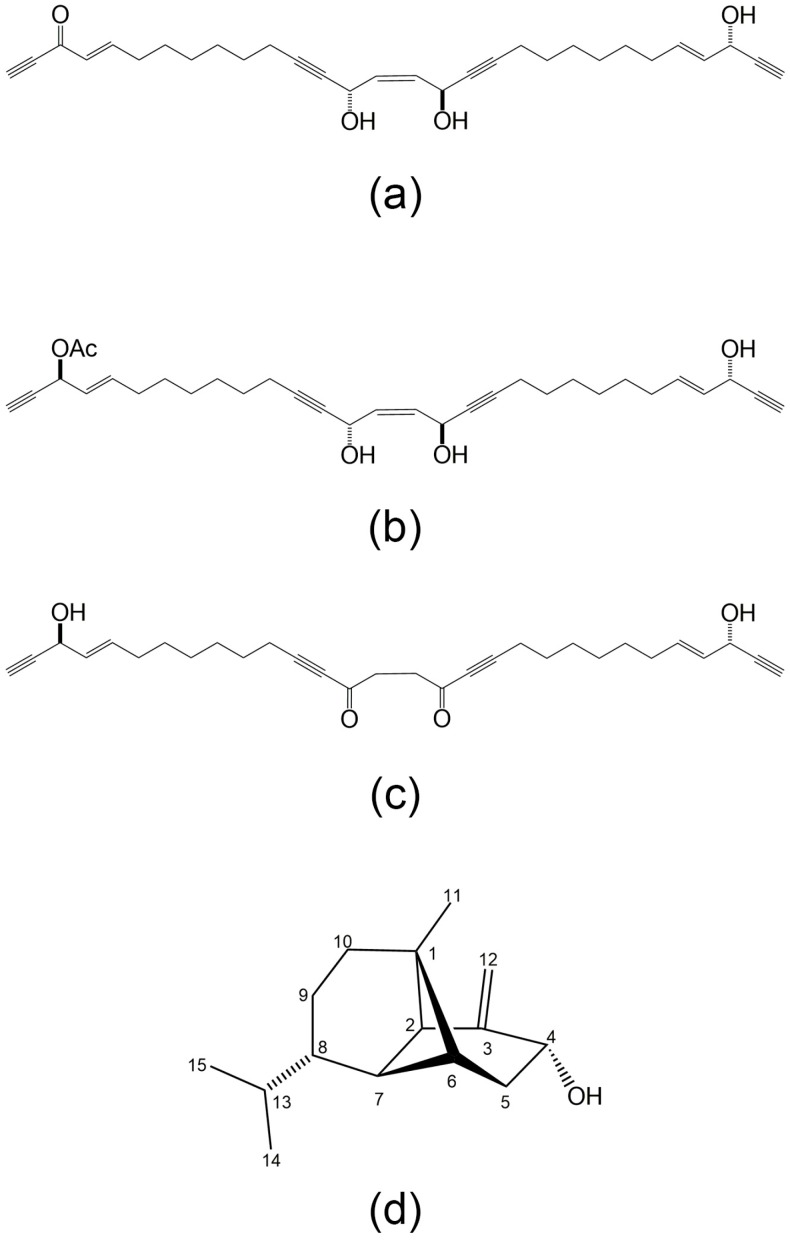
(**a**–**c**) The structures of Adociacetylenes A, C, and D; (**d**) The structure of Lemnalol (8-isopropyl-5-methyl-4-methylene-decahydro-1,5-cyclo-naphthalen-3-ol).

Numerous other compounds or derivatives that are distributed in marine organisms have shown inhibitory effects on neutrophil adhesion. Adociacetylenes A, C, and D (Figure 6a–c) isolated from sponges of the *Adocia* sp. were revealed to have inhibitory activity in the endothelial cell-neutrophil leukocyte adhesion assay in vitro [97]. Lemnalol (8-isopropyl-5-methyl-4-methylene-decahydro-1,5-cyclo-naphthalen-3-ol; Figure 6d), extracted from the soft corals *Lemnalia tenuis* or *Lemnalia cervicorni*, was investigated in a study on monosodium urate (MSU)-induced gouty arthritis in rats [98]. Lemnalol suppressed leukocytes infiltration (primary neutrophils) in synovium injected with MSU. It also reduced inducible NO synthase (iNOS) and COX-2 in synovial tissue. Moreover, some mucin-type glycoproteins purified from the mucous secretions of the starfish *Marthasterias glacialis* and *Porania pulvillus*, as well as the brittlestar *Ophiocomina nigra* were reported to inhibit the adhesion of human neutrophils to cultured human vascular endothelial cells [99].

## 6. Conclusions

Excessive levels of inflammation can lead to a variety of diseases and, therefore, the search for novel anti-inflammatory agents and their investigation has become the focus of biologists and chemists. Neutrophils play a pivotal role in inflammation, and therefore, are considered important targets for anti-inflammatory compounds. Significant progress has been achieved recently in identifying novel anti-neutrophil marine products. However, their mechanisms of action are largely unexplored. In this report, we reviewed numerous marine natural products with mechanisms of action that have been well investigated. In addition, the compounds listed in Table 1, whose molecular targets have not been fully elucidated, may offer excellent opportunities to researchers who are interested in investigating the intracellular molecular mechanisms of compounds in neutrophils.

During inflammation, activated neutrophils release neutrophil extracellular traps (NETs), which are extracellular networks primarily composed of DNA, granule proteins, and modified histone [100]. In addition to phagocytosis and secretion of antimicrobial materials, NETs are an important machinery of neutrophils used to attack invading microbes and are involved in inflammatory responses [100,101]. Nevertheless, exaggerated NETs formation also contributes to some inflammatory diseases, such as systemic lupus erythematosus [102], diabetes [103], disseminated intravascular coagulation [104], and cardiovascular diseases [105]. Although some drugs have been identified and developed to inhibit NETs, no marine natural product has been discovered to suppress NETs. Marine natural products are a valuable source of bioactive entities and potential drug scaffolds; therefore, we predict future comprehensive investigations can discover marine-based NETs inhibitors.

The chemical and biological diversities of marine products motivated some structure–activity relationship studies of some bioactive classes. Rational design and targeted modification of known bioactive marine natural products could offer biological activities improvement and enhance their therapeutic margin. Structure and ligand-based semisynthetic modifications of the thoroughly investigated anti-inflammatory marine-based entities have the potential to facilitate novel drug discoveries.

In conclusion, inflammation is a process that occurs in various cell types including immune and non-immune cells and has a role in fighting pathogenic or sterile injury. However, excessive inflammation can be deleterious to the body and leads to inflammatory diseases. This review provides an overview of previously conducted experiments that examined the anti-inflammatory effects of marine products in neutrophils, and it is our hope that additional studies investigating the detailed mechanisms of the anti-inflammatory effects of natural or synthetic products would be conducted not only on neutrophils but the entire complex immune system.

## Figures and Tables

**Table 1:** Marine nature products that inhibit reactive oxygen species (ROS) or elastase release (E) in *N*-formyl-methionyl-leucyl-phenylalanine-stimulated neutrophils.

Compound	Sources	Activities *	Ref.
Excavatoid E	*Briareum excavatum*	E	[20]
Excavatoid F	*Briareum excavatum*	E	[20]
Excavatoid L	*Briareum excavatum*	ROS, E	[21]
Excavatoid O	*Briareum excavatum*	ROS, E	[22]
Excavatoid P	*Briareum excavatum*	ROS, E	[22]
Carijoside A	*Carijoa* sp.	ROS, E	[23]
Hirsutocospiro A	*Cladiella hirsuta*	ROS, E	[24]
Hirsutalin N	*Cladiella hirsuta*	E	[25]
Hirsutalin S	*Cladiella hirsuta*	ROS, E	[26]
Krempfielin K	*Cladiella krempfi*	E	[27]
Krempfielin M	*Cladiella krempfi*	E	[27]
Krempfielin N	*Cladiella krempfi*	E	[28]
Krempfielin P	*Cladiella krempfi*	ROS, E	[28]
Krempfielin Q	*Cladiella krempfi*	ROS, E	[29]
Krempfielin R	*Cladiella krempfi*	ROS, E	[29]
6-*epi*-cladieunicellin F	*Cladiella* sp.	ROS, E	[30]
Cladielloide B	*Cladiella* sp.	ROS, E	[31]
Cladieunicellin C	*Cladiella* sp.	ROS	[32]
Cladieunicellin H	*Cladiella* sp.	ROS, E	[33]
(–)-solenopodin C	*Cladiella* sp.	ROS, E	[34]
6-*epi*-Yonarasterol B	*Echinomuricea* sp.	ROS, E	[35]
Echinoclerodane A	*Echinomuricea* sp.	ROS, E	[36]
Echinohalimane A	*Echinomuricea* sp.	E	[37]
7β-Hydroperoxycholesterol	*Eudistoma* sp.	E	[38]
Dihydroxyicosanoids	*Farlowia mollis*	ROS, E	[39]
Methylfarnesylquinone	*Homoeostrichus formosana*	ROS, E	[40]
Frajunolide E	*Junceella fragilis*	ROS, E	[41]
Frajunolide J	*Junceella fragilis*	ROS, E	[41]
Junceellolide K	*Junceella fragilis*	E	[42]
(−)-11β,20β-Epoxy-4-deacetoxyjunceellolide D	*Junceella fragilis*	E	[42]
Junceol E	*Junceella juncea*	ROS	[43]
Junceol F	*Junceella juncea*	ROS	[43]
Junceol G	*Junceella juncea*	ROS	[43]
Junceol H	*Junceella juncea*	ROS	[43]
Klymollin M	*Klyxum molle*	ROS, E	[44]
Lobocrassin B	*Lobophytum crassum*	ROS, E	[45]
Menelloide D	*Menella* sp.	E	[46]
(−)-Hydroxylindestrenolide	*Menella* sp.	ROS	[47]
Pseudoalteromone B	*Pseudoalteromonas* sp.	E	[48]
2β-Acetoxyclovan-9α-ol	*Rumphella antipathies*	ROS, E	[49]
9α-Acetoxyclovan-2β-ol	*Rumphella antipathies*	ROS, E	[49]
Rumphellaoic acid A	*Rumphella antipathies*	E	[50]
Rumphellaone C	*Rumphella antipathies*	ROS, E	[51]
Rumphellol A	*Rumphella antipathies*	ROS, E	[52]
Rumphellol B	*Rumphella antipathies*	ROS, E	[52]
Tortuosene A	*Sarcophyton tortuosum.*	ROS	[53]
Sinularbol B	*Sinularia arborea*	ROS	[54]
Flexibilin B	*Sinularia flexibilis*	E	[55]

* Inhibition of reactive oxygen species (ROS) and elastase (E).

## Abbreviations

The following abbreviations are used in this manuscript:

ROS	reactive oxygen species
FPR	formyl peptide receptor
ALX	lipoxin A4 receptor
PI3K	phosphatidylinositol-3-kinase
MAPK	mitogen-activated kinase
PLC	phospholipase C
PIP2	phosphatidylinositol 4,5-bisphosphate
IP3	inositol 1,4,5-trisphosphate
PKC	protein kinase C
NADPH	nicotinamide adenine dinucleotide phosphate
fMLF	formyl-methionyl-leucyl phenylalanine
JNK	c-Jun *N*-terminal kinase
ERK	extracellular signal-regulated kinase
PMA	phorbol myristate acetate
OKA	okadaic acid
PP	protein phosphatase
PLA2	phospholipase A2
AA	arachidonic acid
5-LO	5-lipoxygenase
COX	cyclooxygenase
5-HPTETE	5′-hydroperoxyeicosatetraenoic acid
5-HETE	5-hydroxyicosatetraenoic acid
PGH2	prostaglandin H
LTl	leukotriene
TX	thromboxane
PLS	polysaccharide
Nitric oxide	NO
fCS	fucosylated chondroitin sulfate
MSU	monosodium urate
iNOS	nitric oxide synthase
NETs	neutrophil extracellular traps
